# Renal expression of cytokines and chemokines in diabetic nephropathy

**DOI:** 10.1186/s12882-020-01960-0

**Published:** 2020-07-28

**Authors:** Liliane Silvano Araújo, Bianca Gonçalves Silva Torquato, Crislaine Aparecida da Silva, Maria Luíza Gonçalves dos Reis Monteiro, Ana Luisa Monteiro dos Santos Martins, Marcos Vinícius da Silva, Marlene Antônia dos Reis, Juliana Reis Machado

**Affiliations:** 1 Discipline of General Pathology, Institute of Biological and Natural Sciences of Federal University of Triângulo Mineiro, Praça Manoel Terra, 330, Nossa Senhora da Abadia, Uberaba, Minas Gerais 38025-015 Brazil; 2 Department of Microbiology, Immunology and Parasitology, Institute of Biological and Natural Sciences of Federal University of Triângulo Mineiro, Av. Getúlio Guaritá, n° 130, Nossa Senhora da Abadia, Uberaba, Minas Gerais 38025-440 Brazil

**Keywords:** Diabetic nephropathy, Cytokines, Chemokines, Renal biopsy, Interstitial inflammation

## Abstract

**Background:**

Diabetic nephropathy (DN) is the leading cause of end-stage renal disease worldwide. Inflammatory mediators have been implicated in the pathogenesis of DN, thus considered an inflammatory disease. However, further studies are required to assess the renal damage caused by the action of these molecules. Therefore, the objective of this study was to analyze the expression of cytokines and chemokines in renal biopsies from patients with DN and to correlate it with interstitial inflammation and decreased renal function.

**Methods:**

Forty-four native renal biopsies from patients with DN and 23 control cases were selected. In situ expression of eotaxin, MIP-1α (macrophage inflammatory protein-1α), IL-8 (interleukin-8), IL-4, IL-10, TNF-α (tumor necrosis factor-α), TNFR1 (tumor necrosis factor receptor-1), IL-1β, and IL-6 were evaluated by immunohistochemistry.

**Results:**

The DN group showed a significant increase in IL-6 (*p* < 0.0001), IL-1β (*p* < 0.0001), IL-4 (*p* < 0.0001) and eotaxin (*p* = 0.0012) expression, and a decrease in TNFR1 (*p* = 0.0107) and IL-8 (*p* = 0.0262) expression compared to the control group. However, there were no significant differences in IL-10 (*p* = 0.4951), TNF-α (*p* = 0.7534), and MIP-1α (*p* = 0.3816) expression among groups. Regarding interstitial inflammation, there was a significant increase in IL-6 in scores 0 and 1 compared to score 2 (*p* = 0.0035), in IL-10 in score 2 compared to score 0 (*p* = 0.0479), and in eotaxin in score 2 compared to scores 0 and 1 (*p* < 0.0001), whereas IL-8 (*p* = 0.0513) and MIP-1α (*p* = 0.1801) showed no significant differences. There was a tendency for negative correlation between eotaxin and estimated glomerular filtration rate (eGFR) (*p* = 0.0566).

**Conclusions:**

Our results indicated an increased in situ production of cytokines and chemokines in DN, including IL-6, IL-1β, IL-4, and eotaxin. It was observed that, possibly, eotaxin may have an important role in the progression of interstitial inflammation in DN and in eGFR decrease of these patients.

## Background

Diabetic nephropathy (DN) is a chronic microvascular complication that affects about 20 to 30% of patients with type 2 diabetes mellitus (T2DM). It is considered the leading cause of end-stage renal failure requiring renal replacement therapy [1, 2], although its pathogenesis has not yet been fully elucidated. Immune and inflammatory mechanisms play important role in the development and progression of DN, which is considered a chronic inflammatory disease [3, 4]. Several cells, such as monocytes, macrophages, and lymphocytes, as well as chemokines and cytokines, have been implicated in this process [5, 6]. Among them, it is known that IL-1β, IL-6, TNF-α (tumor necrosis factor-α), IL-8, MIP-1α (macrophage inflammatory protein-1α) are relevant for the development of DN, as they are potentially involved in the onset of disease complications [7–9].

Patients with DN have a predominance of increased plasmatic and urinary levels of inflammatory mediators, both in early and end stages of the disease [9–13]. However, the extent of renal damage caused by immune cell-derived cytokines and chemokines and the importance of such inflammatory mechanisms on the development and progression of DN requires further investigation [14, 15].

Renal biopsies are considered the gold standard for diagnosis of glomerulopathies; however, diabetic patients are only subjected to renal biopsies in cases of atypical clinical courses of DN. Atypical presentations include microalbuminuria without diabetic retinopathy, a rapid decline in eGFR, rapidly increasing proteinuria, a sudden onset of nephrotic syndrome, hematuria, a period of less than 5 years from the diagnosis of diabetes to the onset of nephropathy or signs and symptoms of systemic diseases [16, 17].

However, further studies using this type of samples to investigate mechanisms associated with the expression of inflammatory mediators involved in DN pathogenesis are required, as level of these mediators reflects the direct action of molecules in organs, as well as the relationship with DN. Therefore, this study aims to analyze the expression of cytokines and chemokines such as IL-1β, IL-6, IL-4, IL-10, TNF-α, TNFR1 (tumor necrosis fator receptor-1), IL-8, MIP-1α e eotaxin in renal biopsies from patients with DN and determine its correlation with interstitial inflammation and decreased renal function.

## Methods

### Patients

Forty-four cases of native renal biopsies from adult patients diagnosed with DN were selected from the Renal Pathology Service database of the Federal University of Triângulo Mineiro (UFTM), Uberaba-MG, Brazil, from 1996 to 2018. All cases of DN in patients over 18 years old, with satisfactory samples for analysis and without overlap with other renal diseases were included in the study. Control group (*n* = 23) consisted of kidneys obtained from autopsies of patients older than18 years, with no evidence of infection or previous renal changes. Cases with autolysis, acute tubular necrosis, and congestion with moderate to severe changes were excluded from control group. These samples were obtained from the Pathology Service of the University of São Paulo/Ribeirão Preto. This study was approved by the Ethics and Research Committee of the Federal University of Triângulo Mineiro (no. 3.001.006).

### Renal histopathology

The diagnosis of DN was performed with three samples used for light microscopy (LM), direct immunofluorescence (IF) and transmission electron microscopy (TEM) according to the standard procedures [18].

For LM, 2-μm paraffin sections were stained with hematoxylin and eosin (H&E), Sirius red, methenamine silver, and Masson’s trichrome. LM was used to analyze morphological changes and interstitial inflammation. Interstitial inflammation in DN was scored as score 0 (absence of interstitial inflammation), score 1 (presence of inflammatory infiltrate exclusively around the atrophic tubules) and score 2 (inflammatory infiltrate also occurs in areas other than around atrophic tubules). DN classes were defined according to the pathologic classification of DN [19].

For IF, IgG, IgM, IgA, kappa and lambda light chains, C3 and C1q complement fractions and fibrinogen were detected in 2-μm frozen sections using fluorescein isothiocyanate (FITC)-conjugated antibodies (Dako, Copenhagen, Denmark). IF was used to exclude or identify renal diseases overlapping DN. For TEM, tissue was fixed in 2.5% Karnovsky + 0.2% ruthenium red, then fixed in 2% osmium tetroxide. Next, was dehydrated using a graded series of alcohol and acetone solutions before embedding in Epon 812 resin. Ultra-thin sections of 60 nm were prepared and placed in nickel grids. Sections were then stained with uranyl acetate and examined under a transmission electron microscope (EM-900; Zeiss, Germany) [18]. TEM was used to measure thickness of the glomerular basement membrane (GBM) and to exclude or identify renal diseases overlapping DN. All cases of DN overlapping with other renal diseases were excluded from the study.

### Immunohistochemistry

Immunohistochemistry was performed manually on slides containing 2-μm paraffin-embedded tissue sections using the Novolink non-biotin polymer system (Novolink Polymer Detection System Kit; BL, UK) according to the manufacturer’s recommendations. Specifications of the antibodies used are summarized in Table 1.

**Table 1:** Immunohistochemistry specifications

Primary antibody	Supplier	Clone or code	Antigenic recovery	Concentration
Anti-eotaxin monoclonal antibody	Thermo Fisher Scientific	43,911	Citrate pH 6.0	1:100
Anti-MIP-1α (macrophage inflammatory protein-1α) (CCL3) polyclonal antibody	Thermo Fisher Scientific	PA5–32496	Citrate pH 6.0	1:1600
Anti-IL-4 (interleukin-4) polyclonal antibody	Thermo Fisher Scientific	PA5–25165	Citrate pH 6.0	1:1300
Anti-IL-8 (CXCL8) polyclonal antibody	Thermo Fisher Scientific	PA5–79113	Citrate pH 6.0	1:1400
Anti-IL-10 polyclonal antibody	Thermo Fisher Scientific	PA5–79457	Citrate pH 6.0	1:1200
Anti-TNF-α (tumor necrosis factor-α) monoclonal antibody	Thermo Fisher Scientific	2C8	Citrate pH 6.0	1:1200
Anti-TNF Receptor I (tumor necrosis fator receptor-1) monoclonal antibody	abcam	H398	Citrate pH 6.0	1:300
Anti-IL-1β polyclonal antibody	Novus Biologicals	NBP1–19775	Citrate pH 6.0	1:40
Anti-IL-6 polyclonal antibody	abcam	ab6672	Citrate pH 6.0	1:300

### Quantification of in situ immunostaining

All fields of renal biopsy samples and 40 fields of autopsy kidney fragments, which included glomerular and tubulointerstitial compartments, were analyzed. Immunostained cells showing an intense brownish staining were marked by the observer using the interactive AxionCam ICc 5 (Zeiss, Germany) image analysis system with a 40× objective (final magnification of 1600×). Results were expressed as percentage of marked area compared to total area of the analyzed fields.

### Statistical analysis

A spreadsheet (Microsoft Excel) was created for statistical analysis. Data analysis was performed using GraphPad Prism version 7.0 (GraphPad Software, USA). Normality was tested using Kolmogorov-Smirnov test. In cases of normal distribution and similar variances, parametric ANOVA (F) test was used, followed by *post-hoc* Tukey’s test and Student’s *t*-test (t). In cases of a non-normal distribution, Kruskal-Wallis (H) test was used, followed by *post-hoc* Dunn’s test and Mann-Whitney (U) test. Proportions were compared by Chi-square test (χ^2^). Pearson’s test (r) was used to determine correlations with parametric variables and Spearman’s test (rS) for non-parametric variables. Differences were considered statistically significant when *p* < 0.05.

## Results

### General characteristics of control and DN groups

A total of 44 cases of DN were selected. Subjects had a median age of 53 (23–75) years, most were male (24; 54.55%) and white (34; 77.27%). Subjects in control group (*n* = 23) had a median age of 44 (19–80) years with male predominance (12; 52.17%).

Most DN patients were hypertensive (26; 59.1%), had a mean time since diagnosis of diabetes mellitus of 13.66 ± 6.58 years, and a mean GBM thickness of 750.69 ± 184.03 nm. Laboratorial data of these patients showed mean creatinine levels of 2.22 ± 1.47 mg/dL and mean urea levels of 81.9 ± 41.9 mg/dL. The eGFR was 48.46 ± 34.51 mL/min/1.73m^2^. The mean proteinuria was within the nephrotic range with values of 5.02 ± 4.35 g/day. Regarding DN classification, 27 (61.5%) biopsies were classified as class III, corresponding to the nodular sclerosis class (Kimmelstiel-Wilson nodules), 11 (25%) as class IV, and 6 as classes I, IIa, and IIb, with 2 (4.5%) cases in each class. General characteristics of the patients are summarized in Table 2.

**Table 2:** General characteristics of Control and DN groups

	Control Group (***n*** = 23)	DN Group (***n*** = 44)
**Age (years)**		
*Median (Min-Max)*	44 (19–80)	53 (23–75)
*Mean ± SD*	47 ± 16.57	50.3 ± 13.73^a^
**Gender n (%)**		
*Male*	12 (52.17%)	24 (54.55%)^b^
*Female*	11 (47.83%)	20 (45.55%)
**Color n (%)**		
*White*		34 (77.27%)
*Not white*		4 (9.09%)
*NI*		6 (13.64%)
**SAH n (%)**		
*Yes*		26 (59.1%)
*No*		7 (15.9%)
*NI*		11 (25%)
**Course DM (years)**		
*Mean ± SD*		13.66 ± 6.58
**GBM thickness (nm)**		
*Mean ± SD*		750.69 ± 184.03
**DN Classes (n)**		
*Class I*		2 (4.5%)
*Class IIa*		2 (4.5%)
*Class IIb*		2 (4.5%)
*Class III*		27 (61.5%)
*Class IV*		11 (25%)
**Urea (mg/dL)**		
*Mean ± SD*		81.9 ± 41.9
**Creatinine (mg/dL)**		
*Mean ± SD*		2.22 ± 1.47
**eGFR (mL/min/1.73m**^**2**^**)**		
*Mean ± SD*		48.46 ± 34.51
**Proteinuria (g/day)**		
*Mean ± SD*		5.02 ± 4.35

*DN* Diabetic Nephropathy. *DM* Diabetes Mellitus. *SAH* Systemic arterial hypertension. *GBM* Glomerular basement membrane. *eGFR* Estimated glomerular filtration rate. *SD* Standard deviation.

^a^: t = 0.8607; *p* = 0.3927

^b^: χ^2^ = 0.03417; *p* = 0.8533

Considering the fact that the majority (87%) of cases had advanced disease, we performed analyzes comparing patients with eGFR < 60 mL / min / 1.73m^2^ X patients with eGFR> 60 mL / min / 1.73m^2^ in DN group, as follow: IL-6 (*p* = 0.8129; U = 85); IL-1β (*p* = 0.2197; t = 1.252); IL-4 (*p* = 0.8329; t = 0.2128); TNFR1 (*p* = 0.0996; t = 1.698); IL-10 (*p* = 0.6553; t = 0.4507); TNF-α (*p* = 0.2874; t = 1.082); eotaxin (*p* = 0.0819; t = 1.795); IL-8 (*p* = 0.8882; t = 0.1417) and MIP-1α (*p* = 0.1166; t = 1.614). Thus, we believe the results of this study express the disease mechanism and are not directly related to end-stage renal disease.

### Role of cytokines and chemokines in diabetic nephropathy

**Fig. 1:**
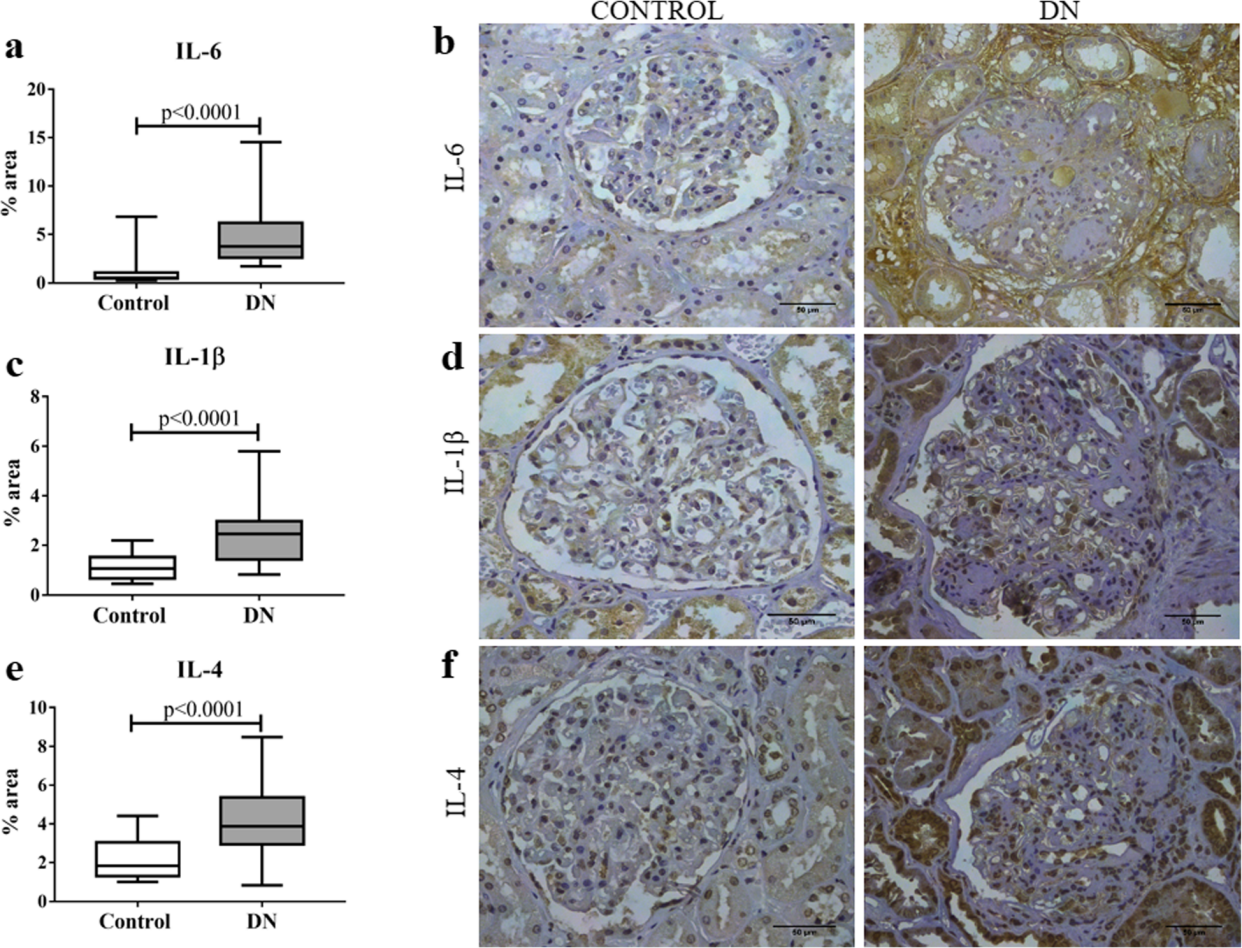
In situ expression of IL-6, IL-1β, and IL-4 in glomerular and tubulointerstitial compartments in patients with diabetic nephropathy (DN) and control group. **a** IL-6 expression in control and DN groups. **b** IL-6 immunostaining in control and DN groups. **c** IL-1β expression in control and DN groups. **d** IL-1β immunostaining in control and DN groups. **e** IL-4 expression in control group and DN groups. **f** IL-4 immunostaining in control and DN groups. Results are expressed as median (min-max). Horizontal lines represent the medians, the bars represent the 25–75% percentiles and the vertical lines represent the percentiles 10–90%

**Fig. 2:**
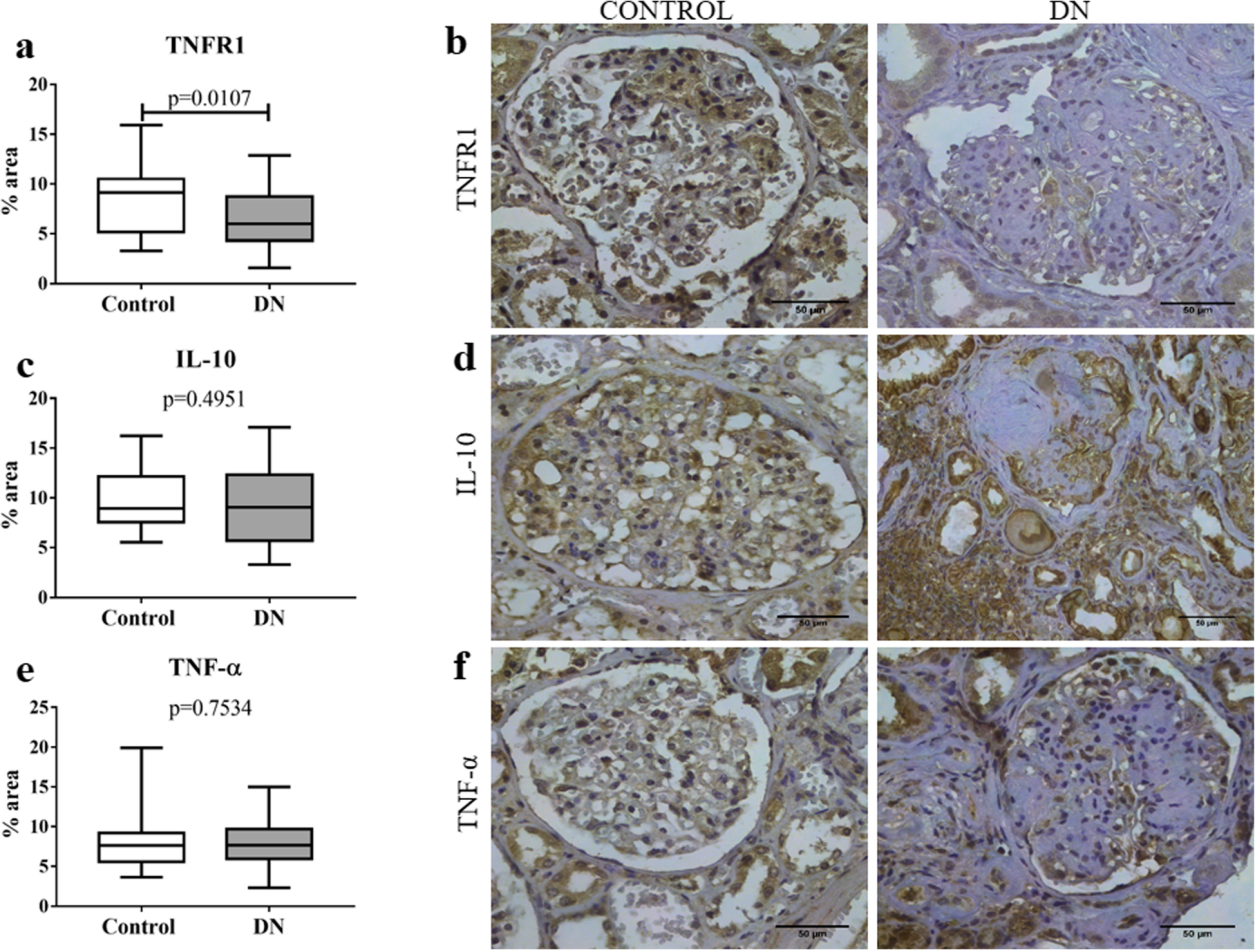
In situ expression of TNFR1, IL-10, and TNF-α in glomerular and tubulointerstitial compartments in patients with diabetic nephropathy (DN) and control group. **a** TNFR1 expression in control and DN groups. **b** TNFR1 immunostaining in control and DN groups. **c** IL-10 expression in control and DN groups. **d** IL-10 immunostaining in control and DN groups. **e** TNF-α expression in control and DN groups. **f** TNF-α immunostaining in control and DN groups. Results are expressed as median (min-max). Horizontal lines represent the medians, the bars represent the 25–75% percentiles and the vertical lines represent the percentiles 10–90%

Expression profile of inflammatory cytokines and chemokines was analyzed in patients with DN and this group showed a significant increase in IL-6 (*p* < 0.0001; U = 82, Fig. 1a and b), IL-1β (*p* < 0.0001; t = 5.16, Fig. 1c and d), and IL-4 (*p* < 0.0001; U = 182, Fig. 1e and f) and a decrease in TNFR1 (*p* = 0.0107; t = 2.631, Fig. 2a and b) compared to the control group. In contrast, there were no significant differences between groups for the cytokines IL-10 (*p* = 0.4951; t = 0.6862, Fig. 2c and d) and TNF-α (*p* = 0.7534; t = 0.3155, Fig. 2e and f).

**Fig. 3:**
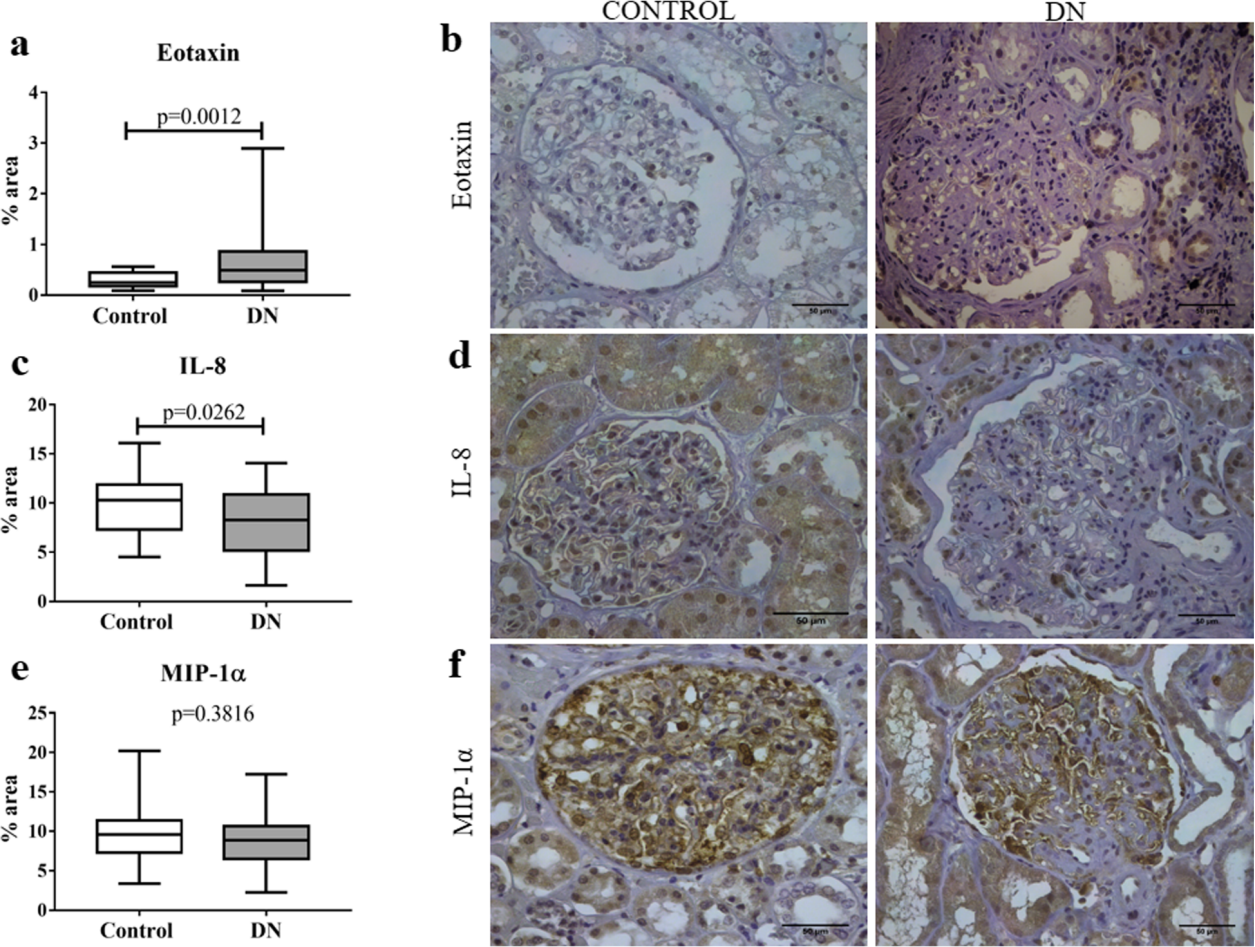
In situ expression of chemokines eotaxin, IL-8, and MIP-1α in glomerular and tubulointerstitial compartments in patients with diabetic nephropathy (DN) and control group. **a** Eotaxin expression in control and DN groups. **b** Eotaxin immunostaining in control and DN groups. **c** IL-8 expression in control and DN groups. **d** IL-8 immunostaining in control and DN groups. **e** MIP-1α expression in control and DN groups. **f** MIP-1α immunostaining in control and DN groups. Results are expressed as median (min-max). Horizontal lines represent the medians, the bars represent the 25–75% percentiles and the vertical lines represent the percentiles 10–90%

Analysis of chemokine expression showed a significant increase in eotaxin (*p* = 0.0012; U = 265.5, Fig. 3a and b) expression and a decrease in IL-8 (*p* = 0.0262; t = 2.275, Fig. 3c and d) expression in DN group compared to control group. However, there were no significant differences between groups for MIP-1α (*p* = 0.3816; t = 0.8811, Fig. 3e and f) expression.

We performed the analysis of cytokine and chemokine expressions in glomerular and tubulointerstitial compartments in DN and control group. The analysis of cytokine expressions in different compartments showed that DN group had a significant increase in IL-6 in glomerular (*p* < 0.0001, U = 77) and tubulointerstitial compartments *(p* < 0.0001, U = 48), IL-1β in glomerular (*p* < 0.0001, U = 179) and tubulointerstitial compartments (*p* < 0.0001, t = 5.325), IL-4 in glomerular (*p* = 0.0014, U = 240) tubulointersticial compartments (*p* < 0.0001, U = 137) and a decrease in TNFR1 in glomerular (*p* = 0.0124, t = 2.574) and tubulointerstitial compartments (*p* = 0.0503, U = 345.5) compared to the control group. However, there were no significant differences between groups for the cytokines IL-10 in glomerular (*p* = 0.3225, t = 0.9972) and tubulointerstitial compartments (*p* = 0.8036, U = 464.5) and TNF-α in glomerular (*p* = 0.2387, U = 396.5) and tubulointerstitial compartments (*p* = 0.2174, U = 392.5). Regarding the expression of chemokines in different compartments, we observed a significant increase in eotaxin in glomerular (*p* = 0.0359, U = 322) and tubulointerstitial compartments (*p* = 0.0007, U = 254) and decrease in glomerular IL-8 (*p* = 0.0127, t = 2.563) in the DN group compared to the control group. However, there was no significant difference between groups for tubulointerstitial IL-8 (*p* = 0.1831, U = 395) and MIP-1α in glomerular (*p* = 0.1487, t = 1.462) and tubulointerstitial compartments (*p* = 0.9349, t = 0.08202).

### Relation of cytokines and chemokines in interstitial inflammation in diabetic nephropathy

**Fig. 4:**
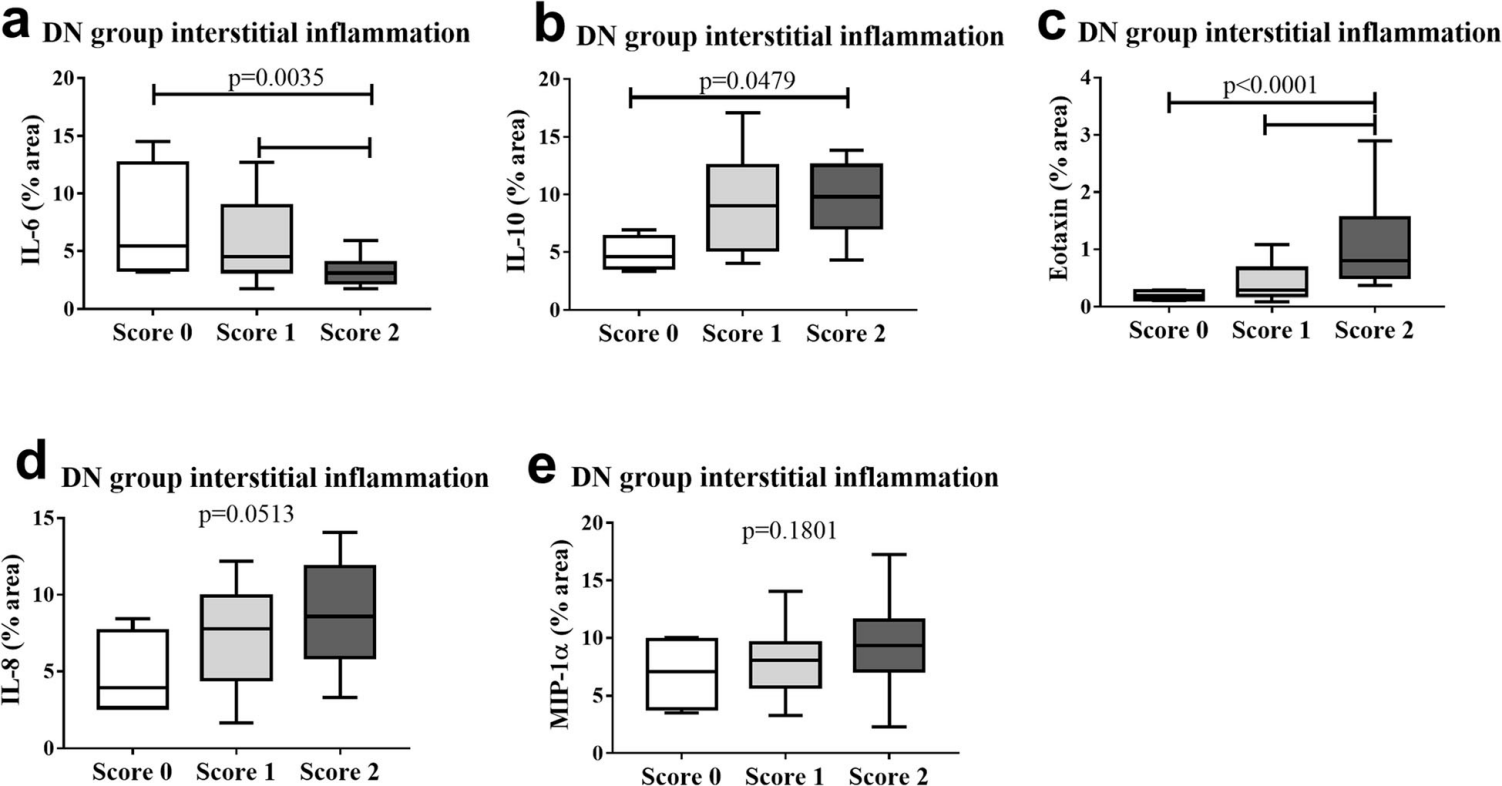
Comparison between in situ cytokine and chemokine expression and interstitial inflammation in patients with diabetic nephropathy (DN). Score 0 (*n* = 4), score 1 (*n* = 19) and score 2 (*n* = 20). **a** IL-6 expression in cases classified as scores 0, 1, and 2 for interstitial inflammation in DN group. **b** IL-10 expression in cases classified as scores 0, 1, and 2 for interstitial inflammation in DN group. **c** Eotaxin expression in cases classified as scores 0, 1, and 2 for interstitial inflammation in DN group. **d** IL-8 expression in cases classified as scores 0, 1, and 2 for interstitial inflammation in DN group. **e** MIP-1α expression in cases classified as scores 0, 1, and 2 for interstitial inflammation in DN group. Results are expressed as median (min-max). Horizontal lines represent the medians, the bars represent the 25–75% percentiles and the vertical lines represent the percentiles 10–90%

After determining the cytokine and chemokine expression profile in DN, we analyzed how these inflammatory mediators could be related to the interstitial inflammation of this disease. There was a significant increase in IL-6 in scores 0 and 1 compared to score 2 (*p* = 0.0035; F = 6.592, Fig. 4a) and a significant increase in IL-10 in score 2 compared to score 0 (*p* = 0.0479; F = 3.295, Fig. 4b). For chemokines, there was a significant increase in eotaxin in score 2 compared to scores 0 and 1 (*p* < 0.0001; H = 19.19, Fig. 4c), whereas IL-8 (*p* = 0.0513; F = 3.208, Fig. 4d) and MIP-1α (*p* = 0.1801; F = 5.203, Fig. 4e) showed no significant differences between groups.

### Correlation between the estimated glomerular filtration rate (eGFR) and chemokine expression in diabetic nephropathy

**Fig. 5:**
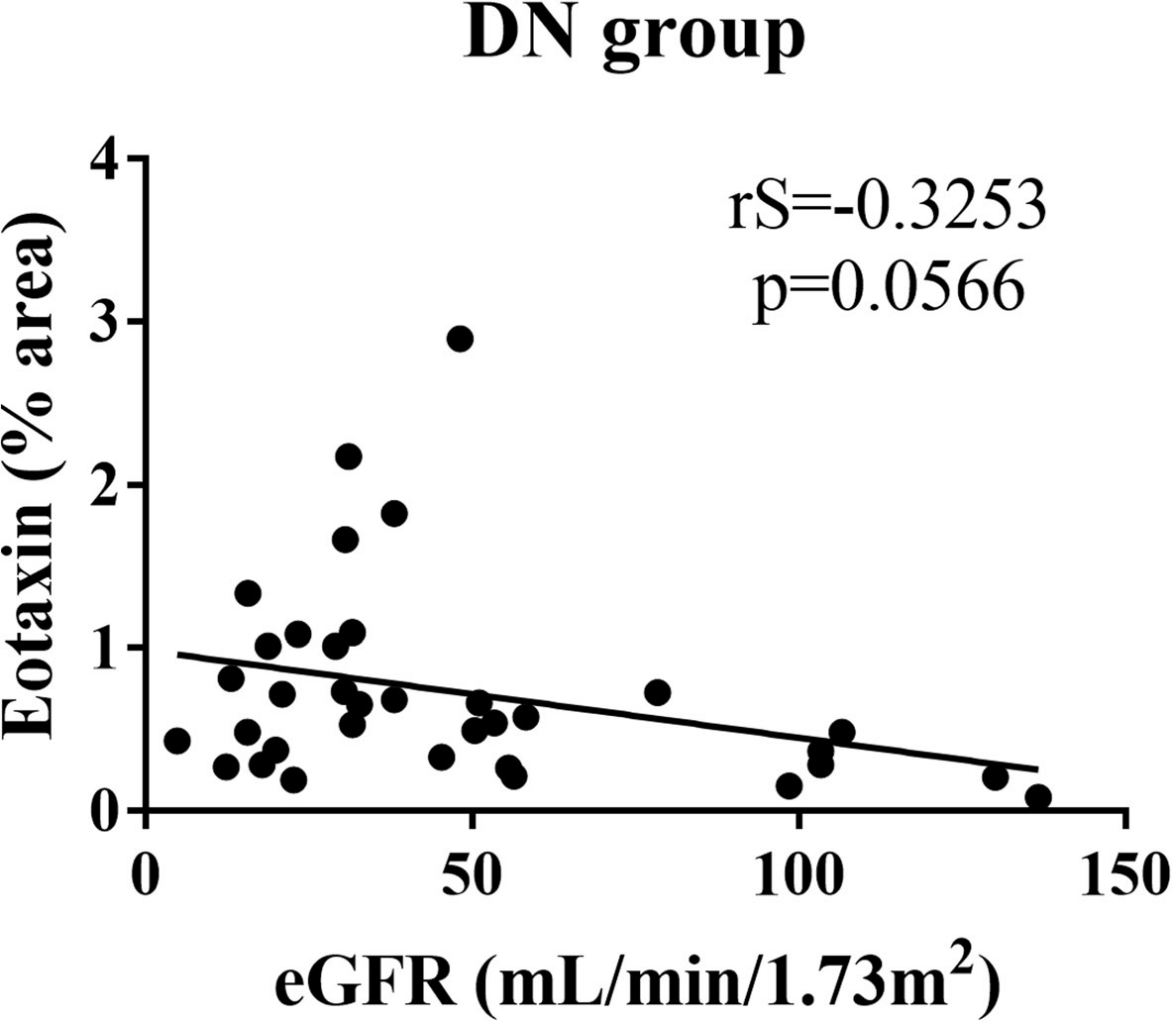
Correlation between estimated glomerular filtration rate (eGFR) and in situ chemokine expression in patients with diabetic nephropathy (DN). Negative and significant correlation trend between eGFR and eotaxin in DN group

As there was a predominant increase in chemokine expression as interstitial inflammation progressed, the potential correlation between chemokine expression and decreased eGFR was analyzed in patients with DN. It was observed that only eotaxin tended to have a negative correlation with eGFR (*p* = 0.0566; rS = − 0.3253, Fig. 5).

### Correlation between proteinuria and cytokines/ chemokine expression in diabetic nephropathy

A possible association between proteinuria and cytokine/ chemokine expression was tested in patients with DN. However, no significant correlation was found between IL-6 (*p* = 0.4123, rS = − 0.1453), IL-1β (*p* = 0.5869, *r* = 0.09509), IL-4 (*p* = 0.2707, *r* = 0.1944), TNFR1 (*p* = 0.0637, *r* = 0.3215), IL-10 (*p* = 0.4248, *r* = − 0.1415), TNF-α (*p* = 0.2167, *r* = − 0.2174), Eotaxin (*p* = 0.1151, rS = 0.2673), IL-8 (*p* = 0.6585, *r* = − 0.0774), MIP-1α (*p* = 0.6225, *r* = − 0.08755) and proteinuria.

## Discussion

This study analyzed in situ expression of cytokines and chemokines in the renal biopsies of patients with DN and related this expression with interstitial inflammation and eGFR to improve our understanding of immune and inflammatory mechanisms that may act directly on kidney and decrease renal function.

Our results showed an increase expression of proinflammatory cytokines IL-6 and IL-1β, as well as of the Th2 cytokine, IL-4 and the chemokine eotaxin in patients with DN. In contrast, TNFR1 and IL-8 expression was reduced in DN. These findings suggest that in DN there may be a simultaneous sharing of cytokine actions of the innate and acquired immune response, associated with the increase of a potent eosinophilic chemokine. A study with microalbuminuric DMT2 patients demonstrated the activation of innate immunity in glomeruli of patients with DMT2 and early nephropathy and suggested that improved Toll-like receptors 4 (TLR4) signaling, expressed in native renal cells, may contribute to the progression of microalbuminuric for macroalbuminuric nephropathy [20]. In addition, it has already been proposed that the identification of transcriptional networks shared between human and mice glomeruli with DN may have a possible role in pathogenesis, which will allow a previous selection of the mouse model that most mimics human DN pathway under investigation [21].

Eotaxin expression may play an important role in DN interstitial inflammation, as its expression was increased exclusively in score 2, in which interstitial inflammation is related to areas other than interstitial fibrosis and tubular atrophy (IFTA) and represents a more severe condition. On the other hand, IL-6 expression was higher in scores 0 and 1 compared to score 2, whereas IL-10 expression was higher in score 2 compared to score 0. Possibly, increase IL-10 expression in score 2 may be affecting IL-6 expression through its pro-fibrotic and anti-inflammatory action.

In addition to the relationship between eotaxin increased expression and interstitial inflammation in patients with DN, there is a correlation between eotaxin expression and decreased eGFR. Therefore, eotaxin on DN may influence both interstitial inflammation and eGFR, indicating its possible role in DN pathogenesis.

Kidney cells (endothelial, mesangial, epithelial, and tubular) are able to synthesize diferente cytokines and chemokines according to the cell and stimuli. Cytokines, chemokines, growth factors, adhesion molecules, nuclear factors, and immune cells, such as monocytes, lymphocytes, and macrophages, have been previously demonstrated to be implicated in DN pathogenesis [22–24]. In this study, patients with DN showed increased expression of IL-6, IL-1β, IL-4 and eotaxin, and decreased expression of TNFR1 and IL-8 both in glomerular and in tubulointerstitial compartment. Thus, it was observed that cells from both renal compartments may be involved in DN pathogenesis and, in this study, the expression of the analyzed cytokines and chemokines was similar in different renal compartments.

IL-1β and IL-6 are among the cytokines that play an important role in DN pathogenesis, affecting renal resident and infiltrating cells. IL-1β induces the expression of the intercellular adhesion molecule 1 (ICAM-1) via mesangial and tubular cells and increases vascular permeability and chemokines expression, resulting in proliferation and synthesis of extracellular matrix (ECM) in glomerular mesangium [25]. IL-6 acts on mesangial cell proliferation and promotes ECM synthesis and GBM thickening, in addition to affecting vascular permeability and facilitating neutrophil infiltration into tubulointerstitium, leading to DN progression [24, 26]. Studies using experimental DN models have shown a correlation between increased renal expression of IL-1β and increased expression of chemotactic factors and adhesion molecules [27, 28]. Previously, T2DM patients with DN were found to show an increased production of IL-6, which was associated with GBM thickness and was considered a strong marker of renal function decline [29]. As GBM thickening is the earliest morphological alteration in DN associated with increased IL-6 production, it is possible that increased in situ IL-6 expression occurs from the early stages of DN, whereby patients show increased expression even without interstitial inflammation (score 0) or in score 1. Moreover, actions associated with increased IL-1β and IL-6 expression promote greater cell infiltration in the kidney, which may exacerbate the inflammatory process and lead to impaired renal function.

Studies have reported that serum IL-10 levels are elevated in T2DM patients with DN and that there is a positive correlation between IL-10 and albuminuria [30–32]. It has been shown that mononucleated cells are able to adopt an anti-inflammatory phenotype in tissue repair process later in the course of inflammation, which is believed to occur after exposure to IL-10. These cells eliminate cellular and matrix debris and generally promote the resolution of renal inflammation, stimulating renal tubular cell proliferation and angiogenesis [33]. Increased IL-10 production most probably represents a compensatory mechanism due to proinflammatory cytokines increased expression and is a negative regulator of inflammation, which corroborates our findings.

Although IL-4 stimulates ECM synthesis through glomerular mesangial and epithelial cells, DN patients were found to have low serum levels of IL-4 [9]. Furthermore, no significant differences were found in this cytokine serum levels comparing patients with and without DN [14]. However, our results show that in situ expression of IL-4 is increased in patients with DN. The main morphological alteration associated with DN is progressive ECM accumulation, which may account for increased IL-4 expression and suggests that the action of this cytokine in promoting ECM synthesis is more effective in situ than systemically.

Studies with T2DM patients have shown that only TNFR1 and TNFR2 receptors are associated with a risk of end-stage renal disease, wherein elevated serum levels of TNFR1 are associated with DN [34] and decreased renal function [35, 36]. TNFR1 is mainly present in glomerular and endothelial cells of the peritubular capillaries [37]. High serum levels of TNFR1 have been associated with global sclerosis, increased ECM, decreased glomerular filtration, and foot process effacement in T2DM patients [38]. Although in vitro-activated TNFR1 induces tissue damage via proinflammatory signals and/or cell death [39], the mechanisms associating TNF receptors with DN remain unknown [38, 40]. However, it has been shown that glomerular and tubular TNFR1 expression is not associated with a loss of renal function nor with any clinical parameters in DN patients [41]. Our results showed decreased in situ expression of TNFR1 in DN, which suggests that elevated serum levels of TNFR1 may be mostly implicated in DN progression.

Eotaxin is a CC chemokine that is specially chemotactic for eosinophils, from the activation of its CCR3 receptor, and is secreted by endothelial cells, macrophages, fibroblasts, and smooth muscle cells [42]. Roy et al. showed that eotaxin could be used as an independent predictor of renal failure. However, the relationship between an increased eotaxin plasma concentration and the progression to renal failure in diabetic patients remains poorly understood [43]. Elevated levels of urinary eotaxin are associated with prolonged hyperglycemia and microalbuminuria in T2DM patients [44]. In kidneys, eotaxin has been reported to contribute to renal interstitial eosinophilia; however, these results do not refer to DN [45].

The slow and continuous decline of renal function is associated with progressive tubulointerstitial damage and renal fibrosis, which is characterized by accumulation of leukocytes, fibroblasts, EMC and tubular atrophy [2, 46]. Accumulation of macrophages and lymphocytes in interstitium is critical for tubular and interstitial damage, since these cells are the main sources of proinflammatory and pro-fibrotic cytokines [47, 48]. Eotaxin is a potent chemoattractant chemokine and/or activator of eosinophils but may also be involved in the regulation of other cells. In atherosclerosis, smooth muscle cells express eotaxin and macrophages and mast cells express the CCR3 receptor, suggesting that eotaxin and its receptor contribute to recruitment and activation of inflammatory cells in ateroma [49]. It was also observed that Th2 lymphocytes, neutrophils, and bronchial endothelial cells also express the CCR3 receptor, suggesting the potential role of eotaxin in the non-eosinophilic inflammatory process [50, 51].

Macrophages are the main inflammatory cells involved in renal damage, the accumulation of which is correlated with DN severity [5, 52, 53] and mesangial expansion [54]. Mast cells also infiltrate tubulointerstitial compartment and release inflammatory mediators and proteolytic enzymes. The intensity of macrophage infiltration and the extent of mast cell degranulation has been previously associated with the level of tubulointerstitial inflammation and with decreased eGFR in DN [55]. Thus, we suggest that the increased in situ expression of eotaxin may be related to its contribution to recruitment and activation of cells other than eosinophils, which strongly promotes further infiltration and accumulation of these cells in kidneys. This may also account for the decreased in situ expression of IL-8 found in patients studied here. Eotaxin action associated with inflammatory cytokines role may worsen the inflammatory process and impair renal function, which corroborates our findings and suggests that eotaxin exerts an in situ role in DN pathogenesis.

In this study, most cases had advanced disease and we recognize this is a limitation of the study, clarified earlier. However, although 87% of our cases were in stage 3–4 of DN, this profile is expected in studies based on renal biopsies samples diagnosed with DN without overlapping with other non-diabetic renal disease [56], due to the indications of the procedure itself. Studies with DN patients showed that severity of glomerular and interstitial lesions had a significant impact on renal prognosis and could be used as independent risk factors for progression of DN [57, 58].

Therefore, our findings indicate that in situ expression analysis of cytokines and chemokines, especially eotaxin, could be used to assist in analysis of renal function impairment based on the analysis of interstitial inflammation developed in patients with DN.

## Conclusions

Our results show that in situ expression of cytokines and chemokines, including IL-6, IL-1β, IL-4 and eotaxin, is increased in patients with DN. It was observed that, possibly, eotaxin may have an important role in progression of interstitial inflammation in DN and in the decrease of eGFR of these patients.

## Data Availability

The datasets used and/or analysed during the current study are available from the corresponding author on reasonable request.

## Abbreviations

DN: Diabetic nephropathy
eGFR: Estimated glomerular filtration rate
ECM: Extracellular matrix
F: ANOVA test
FITC: Fluorescein isothiocyanate
GBM: Glomerular basement membrane
H: Kruskal-Wallis test
H&E: Hematoxylin and eosin
ICAM-1: Intercellular adhesion molecule 1
IF: Direct immunofluorescence
IFTA: Interstitial fibrosis and tubular atrophy
Ig: Immunoglobulin
IL: Interleukin
LM: Light microscopy
MIP-1α: Macrophage inflammatory protein-1α
nm: Nanometers
r: Pearson test
rS: Spearman test
SAH: Systemic arterial hypertension
SD: Standard deviation
t: Student’s t test
T2DM: Type 2 diabetes mellitus
TEM: Transmission electron microscopy
TNF-α: Tumor necrosis factor-α
TNFR1: Tumor necrosis fator receptor-1
TNFR2: Tumor necrosis fator receptor-2
U: Mann Whitney test
χ2: Chi square test
